# Relationship of coregulator and oestrogen receptor isoform expression to *de novo* tamoxifen resistance in human breast cancer

**DOI:** 10.1038/sj.bjc.6600654

**Published:** 2002-12-02

**Authors:** L C Murphy, E Leygue, Y Niu, L Snell, S-M Ho, P H Watson

**Affiliations:** Manitoba Institute of Cell Biology, University of Manitoba, Faculty of Medicine, Winnipeg, Manitoba, Canada, R3E OW3; Department of Biochemistry and Medical Genetics, University of Manitoba, Faculty of Medicine, Winnipeg, Manitoba, Canada, R3E OW3; Department of Pathology, University of Manitoba, Faculty of Medicine, Winnipeg, Manitoba, Canada, R3E OW3; Department of Surgery, University of Massachusetts Medical School, Worchester. Massachusetts, MA 01655, USA

**Keywords:** SRA, AIB1, ROA, coregulators, OR isoforms, human breast cancer, tamoxifen

## Abstract

This study addresses the hypothesis that altered expression of oestrogen receptor-beta and/or altered relative expression of coactivators and corepressors of oestrogen receptors are associated with and may be mechanisms of *de novo* tamoxifen resistance in oestrogen receptor positive breast cancer. All cases were oestrogen receptor +, node negative, primary breast tumours from patients who later had no disease progression (tamoxifen sensitive) or whose disease progressed while on tamoxifen (tamoxifen resistant). Using an antibody to oestrogen receptor-beta that detects multiple forms of this protein (total) but not an antibody that detects only full-length oestrogen receptor-beta 1, it was found that high total oestrogen receptor beta protein expressors were more frequently observed in tamoxifen sensitive tumours than resistant tumours (Fisher's exact test, *P*=0.046). However, no significant differences in the relative expression of oestrogen receptor β2, oestrogen receptor β5 and full-length oestrogen receptor β1 RNA in the tamoxifen sensitive and resistant groups were found. Also, when the relative expression of two known coactivators, steroid receptor RNA activator and amplified in breast cancer 1 RNA to the known corepressor, repressor of oestrogen receptor activity RNA, was examined, no significant differences between the tamoxifen sensitive and resistant groups were found. Altogether, there is little evidence for altered coregulators expression in breast tumours that are *de novo* tamoxifen resistant. However, our data provide preliminary evidence that the expression of oestrogen receptor β protein isoforms may differ in primary tumours of breast cancer patients who prove to have differential sensitivity to tamoxifen therapy.

*British Journal of Cancer* (2002) **87**, 1411–1416. doi:10.1038/sj.bjc.6600654 www.bjcancer.com

© 2002 Cancer Research UK

## 

The ability of anti-oestrogens such as tamoxifen to compete with oestrogens for binding to OR and to antagonise their mitogenic action provides the basic rationale for endocrine therapy and prevention (for a review see (Osborne, 1998b) in breast cancer. Adjuvant tamoxifen post-operative therapy reduces the number of recurrences and prolongs survival in women whose primary tumours are oestrogen receptor (OR) positive (Group, 1998). However, even though OR level is considered a marker for predicting the likelihood of responding to adjuvant hormonal therapies, some patients, whose primary tumours are OR positive do not respond to tamoxifen treatment. Such apparent *de novo* tamoxifen resistance does not depend upon the level of OR within the primary tumour. As well many of those patients whose disease initially responds to tamoxifen, progress while still under treatment having acquired resistance and this occurs despite continued expression of OR. Thus suggesting other components of the oestrogen signalling pathway may be altered. Recent observations using laboratory models (Hall and Mcdonnell, 1999; Lanz *et al*, 1999; Mckenna *et al*, 1999; Montano *et al*, 1999) have demonstrated that altered levels of OR isoforms and/or alteration of expression of coactivators and corepressors can deregulate oestrogen and antioestrogen activity in target cells, suggesting the hypothesis that altered levels of OR isoforms and/or coregulators *in vivo* could be a mechanism of tamoxifen resistance. Previously we have demonstrated that the relative expression of ORα/ORβ as well as the relative expression of some OR coactivators to corepressors is significantly altered during breast tumourigenesis *in vivo* (Leygue *et al*, 1998; Murphy *et al*, 2000). Furthermore, since these alterations parallel the marked changes in oestrogen action that accompany breast tumourigenesis, they may have a role in this process. To explore the hypothesis that such changes could underlie *de novo* tamoxifen resistance *in vivo,* the expression of OR isoforms, two known coactivators (steroid receptor RNA activator (SRA), (Lanz *et al*, 1999) and amplified in breast cancer-1 (AIB1) (Anzick *et al*, 1997)) and one corepressor (repressor of oestrogen receptor activity, repressor of oestrogen receptor activity (ROA) (Montano *et al*, 1999)) of OR activity have been investigated in primary breast tumours from node negative patients whose tumours were OR positive and that subsequently responded or had disease progression while on adjuvant tamoxifen therapy.

## MATERIALS AND METHODS

### Human breast tumours

All breast tumour cases used for this study were selected from the NCIC-Manitoba Breast Tumour Bank (Winnipeg, Manitoba, Canada). As previously described (Hiller *et al*, 1996), tissues are accrued to the Bank from cases at multiple centres within Manitoba, rapidly collected and processed to create matched formalin-fixed-embedded and frozen tissue blocks for each case with the mirror image surfaces oriented by coloured inks. The histology of every sample in the Bank is uniformly interpreted by a pathologist in Hematoxylin/Eosin (H&E) stained sections from the face of the paraffin tissue block. This information is available in a computerized database along with relevant pathological and clinical information and was used as a guide for selection of specific paraffin and frozen blocks from cases for this study. For each case, interpretations included an estimate of the cellular composition (including the percentage of invasive epithelial tumour cells and stroma), tumour type and tumour grade (Nottingham score). Steroid receptor status was determined for all cases by ligand binding assay performed on an adjacent portion of tumour tissue. Tumours with oestrogen receptor levels above 3 fmol mg^−1^ of total protein were considered OR positive.

To identify cases that responded divergently to tamoxifen, review of approximately 1000 consecutive cases was undertaken to identify cases that were OR positive, node negative and that had been treated with adjuvant tamoxifen following surgery +/− local radiation. From these the first cohort of 27 cases was selected to include a subset (*n*=13) that had shown progression of disease (either died or alive with recurrent disease, referred to as tamoxifen resistant cases) and a similar control subset (*n*=14) specifically selected to comprise cases with similar lengths of follow-up, OR status, tumour grade and tumour histology, but that had shown no progression of disease (referred to as tamoxifen sensitive cases). The tumour characteristics were: (1) ‘Tam Sensitive’ group median OR was 60.5 fmol mg^−1^ protein (range 6–146 fmol mg^−1^ protein), median PR was 32 fmol mg^−1^ protein (range 8–216 fmol mg^−1^ protein); median grade was five (range 4–8); median age at biopsy was 69 years (range 35–87 years); median follow-up time was 56 months (range 18–79); (2) ‘Tam Resistant’ group median OR was 57 fmol mg^−1^ protein (range 4–136 fmol mg^−1^ protein); median PR was 14 fmol mg^−1^ protein (range 4–288 fmol mg^−1^ protein); median grade was six (range 4–9); median age at biopsy was 67 years (range 49–83 years); median follow-up time was 56 months (range 9–85).

For the RNA studies, frozen tissue corresponding to the blocks for several of the first cohort of older cases used above, were not available. Therefore, after further review of the tumour bank as described above, a second study cohort was selected that also had frozen tissue available. The relevant patient/tumour characteristics were similar to the above cohort, although the follow-up time was shorter: (1) ‘Tam Sensitive’ group (*n*=16) median OR was 37.5 fmol mg^−1^ protein (range 4.4–146 fmol mg^−1^ protein), median PR was 44 fmol mg^−1^ protein (range 13.1–216 fmol mg^−1^ protein); median grade was six (range 4–9); median age at biopsy was 72 years (range 47–87 years); median follow-up time was 39 months (range 13–76); (2) ‘Tam Resistant’ group (*n*=16) median OR was 21.5 fmol mg^−1^ protein (range 5.6–107 fmol mg^−1^ protein); median PR was 14.3 fmol mg^−1^ protein (range 7.8–288 fmol mg^−1^ protein); median grade was six (range 4–9); median age at biopsy was 71 years (range 60–89 years); median follow-up time was 34 months (range 9–63).

### Immunohistochemistry

Immunohistochemistry was performed on serial 5 μm sections from a representative, formalin fixed paraffin embedded archival tissue block from each tumour. Immunohistochemical staining for ORβ was performed using two different primary antibodies. IgYERB503 (a gift from Dr Jan-Ake Gustafson) detects total ORβ isoforms (Horvath *et al*, 2001; Saji *et al*, 2000) and GC17 (a gift from Dr Shuk-Mei Ho) detects only the full-length ORβ (Leav *et al*, 2001). The GC17 polyclonal antibody was raised in rabbits against a peptide sequence in the F domain of the human OR-β receptor (amino acids 449 to 465) and its specificity validated previously (Leav *et al*, 2001). The epitope to which the IgYERB503 antibody is directed is not known, but this polyclonal chicken antibody was raised to an ORβ recombinant protein which was disrupted in the ligand binding domain by insertion of 18 additional amino acids, but was subsequently shown to also recognise the full-length non-inserted ORβ protein (Saji *et al*, 2000). Antibodies were applied using an automated tissue immunostainer (Discovery module, Ventana Medical Systems, Phoenix, AZ, USA), DAB immunohistochemistry kit and bulk reagents that were supplied by the manufacturer. Briefly, the Discovery staining protocol was set to ‘Standard Cell Conditioning’ procedure, followed by 12 h incubation with primary antibody and 32 min incubation with secondary antibody. Concentrations of primary antibodies initially applied to the Ventana instrument were 1 : 200 for IgYERB503 and 1 : 50 for GC17, which translates into final concentrations of 1 : 600 and 1 : 150 after a 1 : 3 dilution with buffer dispensed onto the slide with the primary antibody. Levels of nuclear ORβ expression were scored semi-quantitatively, under the light microscope. Scores were obtained by estimating average signal intensity (on a scale of 0–300) and the proportion of epithelial cells showing a positive signal (0, none; 0.1, less than one tenth; 0.5, less than one half; 1.0 greater than one half). The intensity and proportion scores were then multiplied to give an overall IHC-score. Cases with a score lower than or equal to 100 were considered negative or weakly positive, whereas tumours with scores higher than 100 were classified as positive for ORβ expression (Al-Haddad *et al*, 1999).

### RNA Extraction and RT–PCR conditions

Total RNA was extracted from 20 μm frozen tissue sections (20 sections per tumour) using Trizol™ reagent (Life Technologies, NY, USA) according to the manufacturer's instructions and quantified spectrophotometrically. One μg of total RNA was reverse transcribed in a final volume of 25 μl as previously described (Leygue *et al*, 1996).

### Primers and PCR conditions

#### Coregulators

The primers used were: SRAcoreU primer (5′-AGGAACGCGGCTGGAACGA-3′; sense; positions 35–53, Genbank accession number AF092038) and SRAcoreL primer (5′-AGTCTGGGGAACCGAGGAT-3′; antisense; position 696–678, Genbank accession number AF092038); AIB1-U primer (5′-ATACTTGCTGGATGGTGGACT-3′; sense; positions 110–130, Genbank accession number AF012108) and AIB1-L primer (5′-TCCTTGCTCTTTTATTTG ACG-3′; antisense; positions 458–438, Genbank accession number AF012108); ROA-U primer (5′-CGAAAAATCTCCTCCCCTACA-3′; sense; positions 385–405, Genbank accession number AF150962) and ROA-L primer (5′-CCTGCTTTGCTTTTTCTACCA-3′; antisense; positions 781–761, Genbank accession number AF150962).

Radioactive PCR amplifications for SRA were performed and PCR products analysed as previously described (Leygue *et al*, 1999b) with minor modifications. Briefly, 1 μl of reverse transcription mixture was amplified in a final volume of 15 μl, in the presence of 1.5 μCi of (α-^32^P) dCTP (3000 Ci mmol^−1^), 4 ng μl^−1^ of each primer and 0.3 unit of Taq DNA polymerase (Gibco BRL, Grand Island, NY, USA). For SRA each PCR consisted of 30 cycles (30 s at 94°C, 30 s at 60°C and 30 s at 72°C). PCR products were then separated on 6% polyacrylamide gels containing 7 M urea. Following electrophoresis, the gels were dried and exposed 2 h to a Molecular Imager™-FX Imaging screen (Bio-Rad, Hercules, CA, USA).

PCR amplifications for AIB1 and ROA were performed and PCR products analysed as previously described (Leygue *et al*, 1996) with minor modifications. Briefly, 1 μl of reverse transcription mixture was amplified in a final volume of 20 μl, in the presence of 4 ng μl^−1^ of each primer and 0.3 unit of Taq DNA polymerase (Gibco BRL, Grand Island, NY, USA). For AIB1, each PCR consisted of 30 cycles (30 s at 94°C, 30 s at 55°C and 30 s at 72°C). For ROA each PCR consisted of 30 cycles (30 s at 94°C, 30 s at 57°C and 30 s at 72°C). PCR products were then separated on agarose gels stained with ethidium bromide as previously described (Leygue *et al*, 1996).

### Primers for OR isoforms

ORα-U primer (5′-TGTGCAATGACTATGCTTCA-3′; sense; located in ORα 792–811) and ORα-L primer (5′-GCTCTTCCTCCTGTTTTTA-3′; antisense; located in ORα 940–922). Nucleotide positions given correspond to published sequences of the human ORα cDNA (Green *et al*, 1986). PCR amplifications were performed and PCR products analysed as previously described with minor modifications (Dotzlaw *et al*, 1997). Briefly, 1 μl of reverse transcription mixture was amplified in a final volume of 15 μl, in the presence of 1 μCi (α-^32^P) dCTP (3000 Ci mmol^−1^), 2 ng μl^−1^ of ORα-U/ORα-L and 0.3 unit of Taq DNA polymerase (Gibco BRL, Grand Island, NY, USA). Each PCR consisted of 30 cycles (30 s at 94°C, 30 s at 60°C and 30 s at 72°C).

A previously validated triple primer assay was used to determine the relative expression of ORβ1 and its variant isoforms ORβ2 and ORβ5 (Leygue *et al*, 1999a). Briefly, 1 μl of reverse transcription mixture was amplified in a final volume of 15 μl, in the presence of 1 μCi of (α-^32^P) dCTP (3000 Ci mmol^−1^), 4 ng μl^−1^ of each primer (ORβ1U, ORβ1L and ORβ2L) and 0.3 unit of Taq DNA polymerase (Gibco BRL, Grand Island, NY, USA).

All OR PCRs consisted of 30 cycles (30 s at 94°C, 30 s at 60°C, and 30 s at 72°C). PCR products were then separated on 6% polyacrylamide gels containing 7 M urea. Following electrophoresis, the gels were dried and autoradiographed. Three independent PCRs were performed.

#### Quantification of SRA and OR RNA expression

Exposed screens were scanned using a Molecular Imager™-FX (Bio-Rad, Hercules, CA, USA) and the intensity of the signal corresponding to SRA or the appropriate OR isoform PCR fragments was measured using Quantity One™ software (Bio-Rad, Hercules, CA, USA). Three independent PCRs were performed. In order to control for variations between experiments, a value of 1 was arbitrarily assigned to the signal of one particular tumour measured in each set of PCR experiments (always the same tumour) and all signals were expressed relative to this signal. Levels of SRA was expressed relative to ROA (SRA/ROA), AIB1 (SRA/AIB1) or ORα (SRA/ORα) in each individual tumour sample. Levels of ORβ isoforms were expressed relative to other ORβ isoforms shown under statistical analysis and as previously described (Leygue *et al*, 1999a).

#### Quantification of the relative expression of the deleted SRA variant RNA

It has previously been shown that the coamplification of a full-length and a deleted variant SRA (SRA-Del) cDNA resulted in the amplification of two PCR products, the relative signal intensity of which provided a reliable measurement of the relative expression of the deleted variant (Leygue *et al*, 1999b). For each sample, the signal corresponding to the SRA-Del was measured using Quantity One™ software (Bio-Rad, Hercules, CA, USA) and expressed as a percentage of the corresponding core SRA signal. For each case, three independent assays were performed and the mean determined.

#### Quantification of ROA and AIB1 RNA expression

Following analysis of PCR products on prestained agarose gels, signals were quantified by scanning using MultiAnalyst™ (Bio-Rad, Hercules, CA, USA). At least, three independent PCRs were performed. A value of 1 was arbitrarily assigned to the ROA or AIB1 signal of one particular tumour and is the same tumour as described above and all signals were expressed relative to this signal. Levels of AIB1 were expressed relative to ROA (AIB1/ROA) and ORα (AIB1/ORα), and levels of ROA were expressed relative to ORα (ROA/ORα).

#### Statistical analysis

Differences between tamoxifen sensitive and tamoxifen resistant cases were tested using the Mann–Whitney rank sum test, two-tailed. Potential differences in expression between the two groups with respect to each ORβ isoform RNA relative to other ORβ isoform RNA expression (e.g. log ORβ1/total ORβ; log ORβ2/total ORβ; log ORβ5/total ORβ, as previously described (Leygue *et al*, 1999a)), and the relative expressions of coregulators (i.e. logAIB1/ROA; logSRA/ROA; logSRA/AIB1; logAIB1/ORα; logSRA/ORα; logROA/ORα) were determined.

Tumours were classified as low (scores between 0 and 100) and high (150–300) ORβ expressors, and differences between tamoxifen sensitive and tamoxifen resistant cases were tested using Fisher's exact test. Correlation between ORβ protein expression (IHC-score) and tumour characteristics was tested by calculation of the Spearman coefficient *r*.

## RESULTS

### Expression of OR*β* protein in primary human breast tumours from patients who later progressed on tamoxifen treatment or showed no progression on tamoxifen treatment

**Figure 1:**
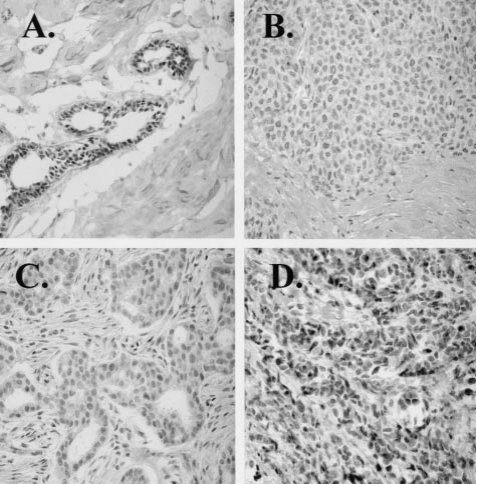
Examples of immunohistochemistry using the GC-17 antibody which only recognises the full-length ORβ1: (**A)** normal human breast tissue; (**B**) ORβ1 negative human breast tumour, H-score=0; (**C**) ORβ1 low expressing human breast tumour, H-score=100; (**D**) ORβ1 high expressing human breast tumour, H-score=150.

ORβ protein was determined immunohistochemically on adjacent sections from each tumour, using two different antibodies. GC-17 is an antibody recognizing an epitope in the C-terminus of full-length ORβ1 (Leav *et al*, 2001). Normal breast tissue was used as a positive control and is shown in Figure 1A

. Examples of staining in human breast tumour sections are shown in Figure 1B–D. Some tumour sections showed no (Figure 1B, full-length ORβ score=0) or low (Figure 1C, full-length ORβ score=100), while others showed strong full-length ORβ signals (Figure 1D, wild-type ORβ score=300). Tumours were classified as low (scores between 0 and 100) and high (150–300) full-length ORβ protein expressors, and differences between tamoxifen sensitive and resistant tumours determined by Fisher's exact test. No significant differences were found.

**Figure 2:**
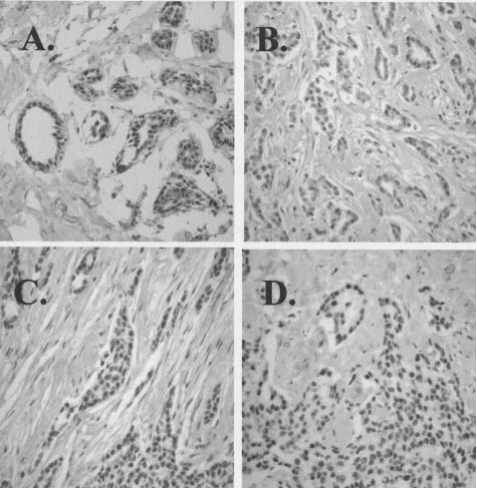
Examples of immunohistochemistry using the IgYERβ503 antibody which recognises most ORβ isoforms: (**A**) normal human breast tissue; (**B**) ORβ negative human breast tumour, H-score=25; (**C**) ORβ low expressing human breast tumour, H-score=100; (**D**) ORβ high expressing human breast tumour, H-score=225.

IgYERbeta503 is an antibody that recognises ligand binding and non-ligand binding ORβ protein isoforms (Horvath *et al*, 2001; Saji *et al*, 2000) and which we refer to as total ORβ protein. Normal breast tissue was used as a positive control and is shown in Figure 2A

. Examples of staining with this antibody in human breast tumour sections are shown in Figure 2B–D. Some sections showed no (Figure 2B, total ORβ score=0) or low (Figure 2C, total ORβ score=100) total ORβ expression whereas others had strong total ORβ protein signal (Figure 2D, total ORβ score=300). Tumours were classified as low and high total ORβ protein expressors, and there was a statistically significant difference in high total ORβ protein between the Tamoxifen sensitive and resistant groups (Fisher's exact test, *P*=0.046). High total ORβ protein expressors were more frequently observed in tamoxifen sensitive tumours than resistant tumours.

Correlation between ORβ protein expression and tumour characteristics was tested by calculation of the Spearman coefficient. A positive correlation between ORβ1 (GC17) protein and progesterone receptor (PR) levels (Spearman *r*=0.44, *P*=0.022) was found when each was examined as continuous variables. When tumours were divided into PR+ (>10 fmol mg^−1^ protein) or PR− (⩽10 fmol mg^−1^ protein) groups there was a significantly higher level of ORβ1 (GC17) protein in PR+ tumours compared to PR− tumours (Mann–Whitney test, *P*=0.0268; median for PR+ tumours=55, range 5–150 and median for PR− tumours=10, range 0–75). As well, there was also a significantly higher level of total ORβ (IgY503) protein in PR+ tumours compared to PR− tumours (Mann–Whitney test, *P*=0.0085; median for PR+ tumours=125, range 25–270 and median for PR− tumours=50 range 0–100).

#### Relative expression of OR*β* isoform RNA in primary human breast tumours from patients who later progressed on tamoxifen treatment or showed no progression on adjuvant tamoxifen

To determine if the differences described above in ORβ protein expression were correlated with differences in ORβ variant isoform RNA expression, we compared the relative expression of ORβ variant RNA to full-length ORβ RNA in the tamoxifen sensitive and resistant groups. Unfortunately, frozen tissue samples corresponding to many of the paraffin blocks from patients in the cohort used for immunohistochemistry were not available. Therefore additional cases selected were selected from the tumourbank as described in Materials and Methods. Using previously validated assays (Leygue *et al*, 1998; 1999a) the relative expression of ORβ2, ORβ5 and full-length ORβ1 RNA in the tamoxifen sensitive and resistant groups was not significantly different.

#### Relative expression of coregulators in primary human breast tumours from patients who later progressed on tamoxifen treatment or showed no progression on tamoxifen treatment

To address the hypothesis that altered relative expression of steroid receptor coactivators and corepressors could underlie altered tamoxifen sensitivity in human breast tumours, and since we previously showed that the relative expression of two coactivators (SRA and AIB1) to a corepressor (ROA) is altered in OR+ breast tumours compared their adjacent normal breast tissue, we chose these coregulators to study. They were measured by RT–PCR in the above tumour cohorts. SRA, AIB1, and ROA mRNAs were detectable in most samples, even though their level of expression differed from one sample to another. Consistent with our previous results (Leygue *et al*, 1999b), an additional fragment, migrating at an apparent size of 459 bp was also observed in most tumours when using SRA specific primers. Sequencing analysis revealed that this band corresponded to a variant form of SRA (referred to as SRA-Del) deleted in 203 bp between positions 155 and 357 (positions given correspond to Genbank accession number AF092038). There were no significant differences between the tamoxifen sensitive and the *de novo* tamoxifen resistant breast cancers in the relative expression of any of the coactivators to corepressor RNA, or in the relative expression of SRA/AIB1 RNA, or in expression of any of these coregulator RNAs relative to ORα or total ORβ RNA expression. As well, there was no significant difference in the relative expression of variant SRA/full-length SRA between the groups either.

#### Tumour characteristics

No statistically significant differences were found between the tamoxifen sensitive and tamoxifen resistant cohorts in any of the tumour characteristics described in the Materials and Methods section except for PR. PR levels were statistically significantly different (*P*=0.044) between the two groups using a Mann–Whitney rank sum test (two sided). PR levels were higher (median PR was 32 fmol mg^−1^ protein; range 8–216 fmol mg^−1^ protein) in the tamoxifen ‘sensitive’ group compared to the tamoxifen ‘*de novo* resistant’ group (median PR was 14 fmol mg^−1^ protein; range 4–288 fmol mg^−1^ protein). This was a consistent finding in both selected cohorts (that used for immunohistochemistry and that used for the RNA study), and provides strong support for differences in oestrogen signalling pathways in these two groups since PR is a marker of OR signal transduction (Horwitz *et al*, 1975; Osborne, 1998a).

## DISCUSSION

We and others have shown that the relative expression of ORα and ORβ is significantly altered during breast tumourigenesis (Leygue *et al*, 1998; Roger *et al*, 2001), and a similar mechanism has been proposed to underlie tamoxifen resistance in breast cancers (Paech *et al*, 1997). The current study shows no significant differences in expression of full-length ORβ (ORβ1) between tamoxifen sensitive and resistant tumours. Interestingly, in this small cohort of tumours when total ORβ expression was examined, there were significantly more high total ORβ expressors in the tamoxifen ‘sensitive’ compared to the ‘resistant’ group. The data suggest the possibility that increased and altered ORβ isoform protein expression may have a role in *de novo* tamoxifen resistance, or at least together with other parameters may provide better markers of endocrine sensitivity. The increased expression of ORβ proteins in the tamoxifen sensitive group is also consistent with recently published data where patients with ORβ positive tumours (determined using an antibody to an N-terminal epitope of the ORβ protein, and defined as nuclear staining in >10% of cancer cells) had a significantly better overall survival than patients with ORβ negative tumours while receiving adjuvant tamoxifen therapy (Mann *et al*, 2001). Both these latter data and those presented currently in this manuscript are in contrast to data showing increased ORβ RNA expression in tamoxifen resistant tumours versus tamoxifen sensitive tumours previously published (Speirs *et al*, 1999). Together these studies suggest that the ORβ status and the nature of ORβ isoforms together with ORα status in human breast cancers may be important biomarkers of endocrine sensitivity, and warrants further study, in larger, prospectively gathered cohorts. The association of increased ORβ isoform expression with tamoxifen sensitivity, suggests a possible mechanistic role, and one possible mechanism may be suggested by several publications which have shown that ORβ isoforms have a modulatory effect on ORα, both in normal tissues (Weihua *et al*, 2000) as well as in cell culture models (Ogawa *et al*, 1998; Hall and Mcdonnell, 1999).

The potential difference between tamoxifen sensitive and resistant groups with respect to ORβ-like proteins, was not correlated with differences in the relative expression of full-length ORβ and two known variants ORβ2 and ORβ5 at the RNA level between the tamoxifen ‘sensitive’ versus the tamoxifen ‘resistant’ groups, however. This may be due to differential regulation of protein versus RNA level or the likelihood that there are other potential ORβ isoforms (known and unknown) expressed in breast tissues in addition to ORβ1, ORβ2 and ORβ5 (Lu *et al*, 1998; Fuqua *et al*, 1999), whose cognate proteins would be detected by the antibody but not measured in the triple primer RT–PCR assay.

Another mechanism for differential tamoxifen sensitivity in OR+ breast tumours could be altered coregulator expression. Although the relative expression of OR coregulators SRA, AIB1 and ROA is altered between normal breast and OR+ breast tumours, there were no significant differences in the ratios of any of the coactivators/corepressors or any of the ratios of these coregulators to ORα RNA levels between primary breast tumours from patients who were later found to be disease free (sensitive) or have disease progression (resistant) while on adjuvant tamoxifen treatment. These data suggest that altered relative expression of these coregulators is unlikely to be a marker of tamoxifen sensitivity in OR+, node negative, primary breast tumours, and unlikely to have a functional role in *de novo* tamoxifen resistance. Although SRA is functional as an RNA molecule, ROA and AIB1 are functional as proteins. Furthermore, other factors can affect protein activity for example phosphorylation in the case of AIB1 (Mora and Brown, 2000) or sequestration by other proteins such as prothymosin-alpha in the case of ROA (Martini *et al*, 2000). Our studies do not exclude differences at the protein and/or activity levels of ROA and AIB1 being involved in *de novo* tamoxifen resistance, nor do they exclude altered expression of these factors having a role in acquired tamoxifen resistance (Lavinsky *et al*, 1998). Altogether, there is little evidence for altered coregulators expression in breast tumours that are *de novo* tamoxifen resistant. However, our data provide preliminary evidence that the expression of ORβ protein isoforms may differ in primary tumours of breast cancer patients who prove to have differential sensitivity to tamoxifen therapy. As well our data support distinct differences in the OR signalling pathways between these two groups of patients since the expression of a known oestrogen responsive gene PR is significantly different between the two groups, the precise mechanisms underlying these differences remain to be elucidated.
